# Maternal and Fetal Complications Associated With Gestational Diabetes Mellitus in a Nicaraguan Tertiary Care Hospital: A Cross-Sectional Study

**DOI:** 10.7759/cureus.81901

**Published:** 2025-04-08

**Authors:** Kenneth Meza Monge, Alma Nubia Rivera Guevara, Natalia Sofia Melara Ruiz, Jose de los Angeles Mendez, Juan-Pablo Idrovo

**Affiliations:** 1 Department of Surgery, Division of Gastrointestinal, Trauma, and Endocrine Surgery, University of Colorado, Anschutz Medical Campus, Aurora, USA; 2 Faculty of Medical Sciences, Universidad Nacional Autónoma de Nicaragua (UNAN) Managua, Managua, NIC; 3 Department of Obstetrics and Gynecology, Bertha Calderón Roque Women's Teaching Hospital, Managua, NIC

**Keywords:** fetal outcomes, gestational diabetes, low-resource settings, macrosomia, maternal complications, preeclampsia

## Abstract

Introduction

Gestational diabetes mellitus (GDM) is a growing public health concern associated with significant maternal and fetal morbidity. Despite global advancements in diagnosis and management, outcomes in low-resource settings such as Nicaragua remain poorly characterized. This study aimed to evaluate the prevalence of maternal and fetal complications associated with GDM and identify factors related to adverse outcomes in a tertiary care hospital in Nicaragua.

Methods

A cross-sectional study was conducted at Bertha Calderón Roque Women's Teaching Hospital in Managua, Nicaragua, from September 1 to December 31, 2017. Seventy women diagnosed with GDM were included through non-random convenience sampling. Data were extracted from clinical records. Descriptive statistics summarized sociodemographic and clinical variables. Exact logistic regression was used to assess associations between maternal factors and adverse outcomes.

Results

Maternal complications occurred in 63% of patients, including preeclampsia (31.4%) and emergency cesarean section (40%). Fetal complications included fetal distress (27.1%) and macrosomia (24.3%). Exact logistic regression identified obesity as significantly associated with cesarean delivery (OR = 3.12, 95% CI: 1.18-8.43, p = 0.021) and preeclampsia (OR = 3.56, 95% CI: 1.24-10.53, p = 0.018). Advanced maternal age (>30 years) was associated with cesarean delivery (OR = 4.21, 95% CI: 1.52-12.14, p = 0.006). Fewer prenatal visits (less than four) were associated with cesarean delivery (OR = 3.97, 95% CI: 1.20-14.33, p = 0.024), preeclampsia (OR = 3.88, 95% CI: 1.22-12.93, p = 0.022), fetal distress (OR = 3.45, 95% CI: 1.07-11.63, p = 0.038), and macrosomia (OR = 3.22, 95% CI: 1.01-10.94, p = 0.048). Although individual complications were common, 98.6% of mother-infant pairs had favorable overall clinical outcomes without severe maternal or neonatal morbidity.

Conclusions

Women with GDM in Nicaragua experience high rates of maternal and neonatal complications, associated with potentially modifiable factors, including obesity, inadequate prenatal care, and advanced maternal age. These findings suggest a need for enhanced screening, improved antenatal coverage, and national clinical guidelines adapted to resource-limited settings.

## Introduction

Gestational diabetes mellitus (GDM) is a growing global health concern, affecting approximately 14% of pregnancies worldwide [1]. Characterized by glucose intolerance first detected during pregnancy, GDM significantly increases the risk of maternal complications such as gestational hypertension and preeclampsia, as well as neonatal adverse outcomes, including macrosomia, neonatal hypoglycemia, and respiratory distress [2]. Advancements in diabetes care have improved outcomes in high-income settings. However, many low-resource regions, including Nicaragua, continue to face substantial challenges in early detection, management, and prevention of GDM-related complications.

In Nicaragua, diabetes is the second most common chronic disease [3]. Although the estimated prevalence of GDM is 13.9%, reliable national data remain scarce due to limited routine screening [1]. Healthcare access is further complicated by geographic and economic barriers, with many rural women unable to attend the recommended number of antenatal visits [4]. This gap underscores the urgent need for improved surveillance and maternal care strategies tailored to resource-constrained settings.

Recent advancements in GDM screening and management have introduced continuous glucose monitoring (CGM) and telemedicine-based interventions as promising alternatives to traditional glucose testing [5]. CGM provides real-time glycemic data, improving the detection of fluctuations that contribute to adverse pregnancy outcomes. Meanwhile, telemedicine has emerged as an effective tool for enhancing glycemic control and reducing cesarean section rates by enabling frequent monitoring and remote consultations [6]. These innovations could offer viable solutions for regions with limited access to specialized prenatal care, such as Nicaragua, though implementation challenges remain substantial.

Recognizing these advancements, global organizations have updated their clinical guidelines to reflect the latest evidence-based practices in GDM detection and care. The World Health Organization (WHO) now recommends universal screening at 24-28 weeks of gestation using a 75 g oral glucose tolerance test (OGTT) to standardize diagnosis globally [7]. Similarly, the American Diabetes Association reinforces early risk assessment, postpartum follow-up, and integration of advanced monitoring technologies to prevent disease progression [8]. These recommendations highlight the shift toward proactive metabolic control and comprehensive postnatal care, aiming to reduce both immediate pregnancy complications and the long-term risk of type 2 diabetes in affected mothers. However, despite these updates, implementation in resource-limited settings remains a major challenge due to financial constraints and gaps in healthcare infrastructure.

Beyond clinical implications, GDM also poses a significant economic burden on healthcare systems, particularly in low-resource countries. A cost analysis from Mexico estimated that each GDM case incurs an additional $2,900 in immediate medical expenses, contributing to a national burden ranging from $87 million to $827 million annually [9]. Although economic assessments specific to Nicaragua are lacking, the anticipated costs of poor GDM management, such as higher cesarean rates, neonatal intensive care admissions, and future diabetes care, are likely significant. Encouragingly, studies indicate that early screening and intervention are cost-effective strategies, preventing severe complications and reducing long-term healthcare expenditures [10].

Therefore, we conducted this study to analyze the prevalence of maternal and fetal complications associated with GDM in a national tertiary care hospital in Nicaragua and to identify factors associated with adverse outcomes. We hypothesized that women with GDM in Nicaragua would experience higher rates of maternal and fetal complications compared to those reported in high-income countries, and that these complications would be associated with potentially modifiable factors such as prenatal care frequency and maternal obesity. By exploring these associations, this cross-sectional study aims to provide insights into the unique challenges faced by underserved populations and contribute to the global understanding of GDM in low-resource settings.

## Materials and methods

Study design and setting

A cross-sectional descriptive study was conducted from September 1 to December 31, 2017, in the Division of Maternal-Fetal Medicine, Department of Obstetrics and Gynecology at Bertha Calderón Roque Women's Teaching Hospital in Managua, Nicaragua. This tertiary care center is affiliated with the Faculty of Medical Sciences at the National Autonomous University of Nicaragua (UNAN-Managua) and serves as the primary national referral center for high-risk obstetric care.

Study population and sampling

Seventy patients diagnosed with GDM during the study period were included using non-random convenience sampling. Patients were identified through a systematic review of hospital admission records, and those meeting the inclusion criteria were enrolled sequentially. We included women with (1) a confirmed diagnosis of GDM according to national guidelines, (2) complete clinical records, and (3) pregnancies completed at the study hospital. We excluded women with (1) pregestational diabetes, (2) incomplete clinical records, and (3) pregnancies completed at other facilities.

The sample size was limited by the study duration and GDM prevalence at the facility. Though modest, this sample is comparable to other specialized GDM studies in resource-limited settings [11,12] and represents the largest available cohort of women with GDM at this Nicaraguan hospital during the study period.

Diagnostic criteria

GDM was diagnosed following national guidelines established by the Nicaraguan Ministry of Health (MINSA), as outlined in Protocol 077: Management of Obstetric Complications [13]. Screening occurred at three gestational periods: (1) before 24 weeks: diagnosis was based on fasting plasma glucose (≥126 mg/dL), glycosylated hemoglobin (HbA1c) (≥6.5%), or random glucose (≥200 mg/dL). A fasting glucose level between 92 and 126 mg/dL indicates GDM. (2) At 24-28 weeks: a 75 g oral glucose tolerance test (OGTT) was performed. GDM was diagnosed with any of the following: fasting glucose ≥92 mg/dL, one-hour ≥180 mg/dL, or two-hour ≥153 mg/dL. (3) At 32-34 weeks: for patients with risk factors or complications, the OGTT was repeated using the same criteria.

Pregestational diabetes was diagnosed when fasting glucose was ≥126 mg/dL or two-hour post-load glucose was ≥200 mg/dL, confirmed with a second test. These diagnostic criteria are consistent with international guidelines from the International Association of Diabetes and Pregnancy Study Groups (IADPSG), the World Health Organization (WHO), the International Federation of Gynecology and Obstetrics (FIGO), and the American Diabetes Association (ADA) [7,14]. Of the 70 patients included in the study, 18 (25.7%) were diagnosed before 24 weeks, 42 (60%) were diagnosed at 24-28 weeks, and 10 (14.3%) were diagnosed at 32-34 weeks of gestation.

Data collection and variables

Clinical records were systematically reviewed using a standardized data extraction form to collect the following data. (1) Sociodemographic characteristics included maternal age, place of residence, educational level, occupation, family medical history, and personal medical history. (2) Gynecological and obstetric history included the number of pregnancies, deliveries, prenatal care visits, body mass index (BMI) at the start of pregnancy, total weight gain during pregnancy, perinatal history, and mode of delivery. (3) Maternal complications included complications during pregnancy (e.g., urinary infections, gestational hypertension, preeclampsia, and metabolic decompensation), complications during delivery (e.g., shoulder dystocia, postpartum hemorrhage, and emergency cesarean), and complications during the puerperium (e.g., chronic hypertension, hypoglycemia, and diabetic ketoacidosis). (4) Fetal complications included preterm birth, macrosomia, fetal distress, neonatal hypoglycemia, congenital malformations, and respiratory distress syndrome. (5) Maternal and fetal outcomes included delivery outcomes and postnatal evolution of mother and newborn.

Key variables were defined as follows: (1) BMI was categorized according to the WHO standards: normal (18.5-24.9 kg/m²), overweight (25.0-29.9 kg/m²), and obese (≥30.0 kg/m²). (2) Prenatal care: following WHO recommendations, adequate prenatal care was defined as ≥ four visits; inadequate was defined as less than four visits. (3) Maternal weight gain was categorized as <6 kg, 6-11 kg, or >11 kg based on local clinical practice guidelines. (4) Macrosomia was defined as birth weight >4,000 g. (5) Fetal distress was defined as abnormal fetal heart rate patterns requiring intervention. (6) Severe metabolic decompensation was defined as episodes of uncontrolled hyperglycemia requiring inpatient stabilization, often accompanied by ketosis, dehydration, or marked glycemic fluctuations compromising maternal-fetal status. (7) Satisfactory clinical outcome was defined as the absence of severe maternal morbidity (e.g., eclampsia, sepsis, or postpartum hemorrhage requiring transfusion) and neonatal survival without major complications requiring intensive care.

Ethical considerations

This study involved a retrospective review of clinical records without direct patient intervention. In accordance with international ethical standards for observational research, and given the minimal risk nature of the study, individual informed consent was waived. The study was conducted with approval from the hospital administration and the academic department at UNAN-Managua, following their established protocols for medical record-based research.

While Nicaragua lacked a formal IRB structure at the time of the study, we adhered to principles of the Declaration of Helsinki regarding confidentiality and data protection. All patient identifiers were removed during data collection, and results are presented only in aggregate form to protect patient privacy. This approach is consistent with ethical practices for retrospective medical record reviews in settings with limited research infrastructure.

We acknowledge this ethical review limitation as a constraint of conducting research in resource-limited settings and have taken all available measures to ensure participant protection and data security.

Statistical analysis

Data were analyzed using IBM SPSS Statistics version 25 (IBM Corp., Armonk, NY) and SAS 9.4 (SAS Institute, Cary, NC) for the exact logistic regression analyses. Descriptive statistics summarized continuous variables as means with standard deviations, and categorical variables as frequencies and percentages.

Given the modest sample size and risk of sparse data, we employed exact logistic regression rather than conventional maximum likelihood estimation to assess associations between maternal characteristics and adverse outcomes. This approach is specifically designed for small samples and provides more reliable estimates when data are limited. We report odds ratios (ORs) with 95% confidence intervals (CIs) and p-values, with statistical significance defined as p < 0.05.

For comparative analyses of delivery modes and maternal-fetal outcomes, chi-square tests were used. To minimize type I error from multiple comparisons, we considered these analyses exploratory and interpreted them cautiously. Four primary outcomes were pre-specified for the regression analyses: emergency cesarean section, preeclampsia, fetal distress, and macrosomia.

## Results

Sociodemographic characteristics

Nearly half (47.1%) of the patients were over 30 years old, while 28.6% were aged 21-25 years, and 22.9% were 26-30 years old. Adolescents were least represented (1.4%). Most patients had completed high school (65.7%), while 24.3% had only elementary education, and 10% pursued higher education. Urban residents comprised 92.9% of the cohort. The predominant occupation was homemaker (90%), with 5.7% employed and 4.3% unemployed (Table 1).

**Table 1:** Sociodemographic characteristics of women with gestational diabetes mellitus (N = 70).

Variables	Number of patients	Percentage (%)
Age groups		
15-20 years	1	1.4
21-25 years	20	28.6
26-30 years	16	22.9
Over 30 years	33	47.1
Education level		
Elementary school	17	24.3
High school	46	65.7
College/university	6	8.6
Technical education	1	1.4
Residence		
Urban	65	92.9
Rural	5	7.1
Occupation		
Homemaker	63	90.0
Employed	4	5.7
Unemployed	3	4.3

Medical and obstetric history

Family histories included type 2 diabetes (37.1%), hypertension (31.4%), preeclampsia (7.1%), and tuberculosis (5.7%), while 18.6% reported no family medical history (Table 2). More than half of the patients (65.7%) had no notable personal medical history. However, 34.3% had at least one medical condition, primarily obesity (31.4%), prior gestational diabetes (10%), hypertension (7.1%), and preeclampsia (5.7%) (Table 2).

**Table 2:** Family and medical history of women with gestational diabetes mellitus (N = 70).

Variables	Response	Number of patients	Percentage (%)
Family history			
Tuberculosis	Yes	4	5.7
	No	66	94.3
Type 2 diabetes mellitus	Yes	26	37.1
	No	44	62.9
Arterial hypertension	Yes	22	31.4
	No	48	68.6
Preeclampsia	Yes	5	7.1
	No	65	92.9
No family medical history	Yes	13	18.6
	No	57	81.4
Medical history			
Bronchial asthma	Yes	2	2.9
	No	68	97.1
Obesity	Yes	22	31.4
	No	48	68.6
Previous gestational diabetes	Yes	7	10.0
	No	63	90.0
Arterial hypertension	Yes	5	7.1
	No	65	92.9
Previous preeclampsia	Yes	4	5.7
	No	66	94.3
No medical conditions	Yes	24	34.3
	No	46	65.7

Among obstetric histories, 30% were primigravida, 24.3% were bigravida, 15.7% were trigravida, and 30% were multigravida. The majority (42.9%) were multiparous, while 30% were nulliparous. Prior miscarriages were reported in 15.7% of cases. Cesarean history was noted in 34 patients, with most (41.4%) having undergone one procedure. Most women attended four to six prenatal care visits (57.1%), while 21.4% had fewer than four visits. Nutritional status was normal in 42.9% of patients, with obesity present in 31.4%. Weight gain was mostly 6-11 kg (57.1%), with 30% gaining <6 kg and 12.9% >11 kg (Table 3).

**Table 3:** Gynecologic, obstetric, and perinatal history of women with gestational diabetes mellitus (N = 70).

Variables	Categories	Number of patients	Percentage (%)
Number of pregnancies	Primigravida	21	30.0
	Bigravida	17	24.3
	Trigravida	11	15.7
	Multigravida	21	30.0
Pregnancy outcomes	Nulliparous	21	30.0
	Primiparous	19	27.1
	Multiparous	30	42.9
Miscarriages	0	59	84.3
	1	8	11.4
	2	3	4.3
Previous cesarean sections	0	36	51.4
	1	29	41.4
	2	3	4.3
	3	2	2.9
Number of prenatal visits	0	2	2.9
	1-3	15	21.4
	4-6	40	57.1
	≥7	13	18.6
Body mass index (BMI)	Normal (18.5-24.9)	30	42.9
	Overweight (25.0-29.9)	18	25.7
	Obese (≥30.0)	22	31.4
Maternal weight gain	<6 kg	21	30.0
	6-11 kg	40	57.1
	>11 kg	9	12.9

Maternal and fetal outcomes

**Figure 1:**
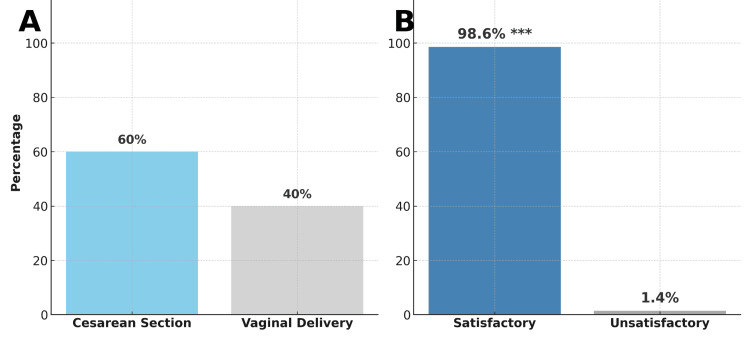
Mode of delivery and maternal-fetal clinical evolution. (A) Illustrates the distribution of delivery modes, with 60% undergoing cesarean section and 40% delivering vaginally. (B) *S*hows maternal-fetal clinical evolution, with 98.6% of cases classified as satisfactory and only 1.4% as unsatisfactory. A satisfactory clinical outcome was defined as the absence of severe maternal morbidity and neonatal survival without major complications requiring intensive care. The observed distribution was significantly skewed toward favorable outcomes (χ² = 66.06, df = 1, p < 0.0001), as indicated by asterisks (***).

Previous perinatal complications were relatively uncommon, with 94.3% having no prior perinatal complications. Cesarean delivery was the predominant mode of birth (60%), while 40% had a vaginal delivery (Figure 1A). Maternal complications affected 63% of patients, primarily preeclampsia (31.4%), emergency cesarean section (40%), and severe metabolic decompensation (14.3%). Infectious complications included vaginal candidiasis (10%) and urinary tract infections (2.9%). Other notable complications were polyhydramnios (8.6%), gestational hypertension (8.6%), and hypoglycemia (4.3%) (Table 4).

**Table 4:** Maternal complications during pregnancy, labor, and puerperium in women with gestational diabetes mellitus (N = 70).

Complications	Response	Number of patients	Percentage (%)
Complications during pregnancy			
Threatened preterm labor	Yes	4	5.7
	No	66	94.3
Labor induction or cesarean section	Yes	10	14.3
	No	60	85.7
Urinary tract infection	Yes	2	2.9
	No	68	97.1
Vaginal candidiasis	Yes	7	10.0
	No	63	90.0
Polyhydramnios	Yes	6	8.6
	No	64	91.4
Preeclampsia	Yes	22	31.4
	No	48	68.6
Gestational hypertension	Yes	6	8.6
	No	64	91.4
Severe metabolic decompensation	Yes	10	14.3
	No	60	85.7
Hypoglycemia	Yes	3	4.3
	No	67	95.7
None	Yes	26	37.1
	No	44	62.9
Complications during labor			
Premature rupture of membranes	Yes	8	11.4
	No	62	88.6
Difficult labor due to fetal size	Yes	2	2.9
	No	68	97.1
Shoulder dystocia	Yes	1	1.4
	No	69	98.6
Neonatal hypoglycemia	Yes	1	1.4
	No	69	98.6
Postpartum hemorrhage	Yes	4	5.7
	No	66	94.3
Emergency cesarean section	Yes	28	40.0
	No	42	60.0
Hypovolemic shock	Yes	1	1.4
	No	69	98.6
None	Yes	25	35.7
	No	45	64.3
Puerperium complications			
Chronic hypertension	Yes	7	10.0
	No	63	90.0
None	Yes	63	90.0
	No	7	10.0

Fetal complications included fetal distress (27.1%) and macrosomia (24.3%). Neonatal asphyxia (5.7%), intrauterine growth restriction (2.9%), and congenital malformations (1.4%) were less common. All pregnancies reached full term, with no preterm births. One-third (32.9%) of neonates had no complications (Table 5).

**Table 5:** Fetal complications in women with gestational diabetes mellitus (N = 70).

Complications	Response	Number of patients	Percentage (%)
Prematurity	Yes	0	0
	No	70	100
Fetal macrosomia	Yes	17	24.3
	No	53	75.7
Fetal distress	Yes	19	27.1
	No	51	72.9
Intrauterine growth restriction	Yes	2	2.9
	No	68	97.1
Neonatal asphyxia	Yes	4	5.7
	No	66	94.3
Congenital malformations	Yes	1	1.4
	No	69	98.6
None	Yes	23	32.9
	No	47	67.1

Overall, 98.6% of mother-infant pairs experienced a satisfactory clinical outcome without severe maternal morbidity or major neonatal complications requiring intensive care, while only 1.4% had an unsatisfactory outcome. This distribution was significantly different from an even distribution (χ² = 66.06, df = 1, p < 0.0001), indicating predominantly favorable clinical outcomes despite the high prevalence of individual complications (Figure 1B).

Exact logistic regression analysis of maternal risk factors and adverse outcomes

Exact logistic regression analyses identified several maternal factors associated with adverse outcomes (Table 6). Women with obesity had significantly higher odds of undergoing a cesarean section compared to non-obese women (OR = 3.12, 95% CI: 1.18-8.43, p = 0.021). Similarly, being older than 30 years was associated with cesarean delivery (OR = 4.21, 95% CI: 1.52-12.14, p = 0.006). Inadequate prenatal care, defined as fewer than four prenatal visits, was also significantly associated with increased odds of cesarean delivery (OR = 3.97, 95% CI: 1.20-14.33, p = 0.024).

**Table 6:** Exact logistic regression analysis of maternal risk factors associated with adverse outcomes. This table summarizes the exact logistic regression models evaluating associations between maternal risk factors and adverse outcomes. Variable outcomes include cesarean section, preeclampsia, fetal distress, and macrosomia. Predictors include obesity, maternal age over 30 years, and inadequate prenatal care frequency (< four visits). The odds ratio (OR) reflects the strength of association between each predictor and outcome; an OR >1 indicates increased odds. The 95% confidence interval (CI) indicates the estimated precision of the OR, and p-values <0.05 denote statistical significance.

Outcome	Predictor	Odds ratio	95% CI	p-value
Cesarean section	Obesity	3.12	1.18–8.43	0.021
Cesarean section	Maternal age over 30 years	4.21	1.52–12.14	0.006
Cesarean section	Inadequate prenatal care (<4 visits)	3.97	1.20–14.33	0.024
Preeclampsia	Obesity	3.56	1.24–10.53	0.018
Preeclampsia	Inadequate prenatal care (<4 visits)	3.88	1.22–12.93	0.022
Fetal distress	Obesity	2.98	1.04–8.92	0.042
Fetal distress	Inadequate prenatal care (<4 visits)	3.45	1.07–11.63	0.038
Macrosomia	Obesity	3.09	1.05–9.45	0.041
Macrosomia	Inadequate prenatal care (<4 visits)	3.22	1.01–10.94	0.048

Preeclampsia was more likely to occur in women with obesity (OR = 3.56, 95% CI: 1.24-10.53, p = 0.018) and in those with fewer prenatal visits (OR = 3.88, 95% CI: 1.22-12.93, p = 0.022).

Regarding fetal complications, obesity was significantly associated with fetal distress (OR = 2.98, 95% CI: 1.04-8.92, p = 0.042), while inadequate prenatal care also increased the odds of fetal distress (OR = 3.45, 95% CI: 1.07-11.63, p = 0.038). Macrosomia was associated with both obesity (OR = 3.09, 95% CI: 1.05-9.45, p = 0.041) and inadequate prenatal care (OR = 3.22, 95% CI: 1.01-10.94, p = 0.048).

## Discussion

This study provides insights into maternal and fetal complications associated with GDM in a resource-limited setting. Conducted at Bertha Calderón Roque Women's Teaching Hospital in Nicaragua, it combines descriptive prevalence data with multivariate exact logistic regression modeling to identify factors associated with adverse outcomes.

Key findings in the local and global context

The majority of patients were over 30 years old, a demographic trend aligned with studies in Peru, China, and among Hispanic women in the US [15-17]. This is expected, as advanced maternal age is a recognized risk factor for GDM and related complications due to increased insulin resistance and vascular changes [18]. Our exact logistic regression analysis confirmed that maternal age >30 years significantly increased the likelihood of cesarean delivery (OR = 4.21), reinforcing age as a clinical indicator requiring additional attention in GDM pregnancies.

Obesity, observed in 31.4% of patients, was consistently associated with multiple adverse outcomes, including cesarean delivery, preeclampsia, fetal distress, and macrosomia in both descriptive analyses and through exact logistic regression. This mirrors findings in high-income countries, where maternal obesity is a well-established contributor to pregnancy complications due to its effects on endothelial function, inflammation, and insulin sensitivity [19-21]. However, in contrast to the US, where structured preconception counseling and multidisciplinary GDM clinics help mitigate risks, patients in Nicaragua may lack access to routine weight monitoring, tailored nutritional advice, or glucose self-monitoring.

A particularly concerning finding is the prevalence of preeclampsia in 31.4% of patients, considerably higher than the approximately 9.6% reported in high-income settings like the US and Europe [22,23]. The association between preeclampsia and obesity, as demonstrated in our regression models, highlights the importance of early screening and preventive strategies, such as the use of low-dose aspirin in high-risk pregnancies, an intervention widely implemented in the US but not yet standard in Nicaragua.

Cesarean delivery occurred in 60% of cases, with 40% being emergency procedures. These rates are higher than those reported in GDM populations in Australia and Canada, where cesarean rates typically range from 19.8% to 33% in women with GDM [24,25]. The high cesarean rate observed in our study may reflect both medical indications and system-level factors such as differences in obstetric practices and decision-making protocols. This finding underscores the need for locally adapted clinical guidelines that address both obstetric management and institutional delivery practices.

Inadequate prenatal care as a cross-cutting risk factor

One of the most consistent findings across all outcomes, including preeclampsia, cesarean delivery, fetal distress, and macrosomia, was the association with fewer than four prenatal visits. This aligns with the WHO recommendations suggesting that a minimum of eight quality antenatal visits can significantly reduce maternal and perinatal mortality [26]. In high-income countries like the US, structured prenatal care facilitates early GDM detection, counseling, and monitoring, while the infrastructure is often fragmented in Nicaragua [7].

The strong association between inadequate prenatal care and adverse outcomes likely reflects multiple interrelated factors. First, limited antenatal visits reduce opportunities for early GDM detection and intervention. Second, women with fewer visits may have less exposure to nutritional counseling and glycemic management education. Third, reduced monitoring may delay recognition of developing complications such as preeclampsia or fetal growth abnormalities. Finally, socioeconomic barriers that limit prenatal care access may coincide with other social determinants of health that negatively impact pregnancy outcomes.

Macrosomia and neonatal asphyxia: elevated rates and contributing factors

Macrosomia was observed in 24.3% of newborns, which exceeds rates reported in some Latin American countries and the 15-20% prevalence typically reported among GDM pregnancies in the United States [27,28]. Several factors likely contribute to this elevated rate in Nicaragua, including delayed or limited glycemic control due to late diagnosis, cultural dietary patterns, minimal access to nutrition counseling, and insufficient glucose monitoring. Our regression analysis confirmed that macrosomia was independently associated with both maternal obesity and inadequate prenatal care, reinforcing the importance of early lifestyle interventions and structured antenatal follow-up.

Neonatal asphyxia, though less prevalent (5.7%), represents a significant outcome. While neonatal asphyxia is a multifactorial condition, excessive fetal growth, common in poorly controlled GDM, can contribute to labor complications such as shoulder dystocia, prolonged delivery, and hypoxic events. Unlike high-income countries, where frequent fetal surveillance and planned early delivery are often used to reduce intrapartum risk, limited prenatal monitoring in Nicaragua may delay recognition of fetal compromise [29,30]. Addressing these gaps through nutritional guidance, accessible glucose monitoring, and standardized weight gain tracking could significantly reduce fetal complications.

Practice and policy implications

These findings suggest several targeted interventions that are feasible within the Nicaraguan health system: (1) implement simplified GDM screening using capillary glucose testing during routine prenatal visits, especially for women >30 years or with obesity. (2) Provide community-based nutritional counseling, including culturally relevant diet planning and portion control for women with or at risk for GDM. (3) Expand antenatal visit coverage by integrating midwives and community health workers into rural and underserved areas to increase prenatal care access. (4) Consider mobile health initiatives to improve visit attendance and self-monitoring using mobile phones, a strategy shown to be effective in other low-resource settings.

From a policy perspective, developing national clinical guidelines for GDM management, adapted to local resource availability, could standardize care and improve outcomes. Additionally, subsidizing basic glucose monitoring supplies through public health programs would address a critical gap in care.

Novel contributions and research impact

This study provides documentation of both descriptive prevalence and multivariable risk patterns for GDM-related outcomes in Nicaragua. The notably higher rates of preeclampsia and cesarean delivery compared to global data highlight specific vulnerabilities in this population and inform the design of locally relevant intervention strategies. Moreover, the consistent associations with inadequate prenatal care provide an evidence base for health system redesign, even in resource-constrained environments.

Limitations

This study has inherent limitations that should be considered when interpreting the results. First, the cross-sectional design precludes establishing causality between GDM and observed maternal-fetal outcomes. While we identified significant associations between risk factors and complications, these relationships should be interpreted as correlational rather than causal.

Second, the sample size of 70, though consistent with similar studies in resource-limited settings, limited the statistical power for detecting associations, particularly for less common outcomes. To address this limitation, we employed exact logistic regression methods specifically designed for small samples, which provide more reliable estimates than conventional approaches when data are limited.

Third, reliance on clinical records may introduce information bias through incomplete documentation or variable classification of outcomes. We attempted to mitigate this by using standardized data extraction forms and clearly defined outcome criteria, but some degree of misclassification remains possible.

Fourth, this study's single-center, hospital-based design potentially introduces selection bias, as it includes only women who delivered at a tertiary referral center. This may overrepresent severe cases and underrepresent women with well-controlled GDM managed in outpatient settings or those with limited healthcare access who delivered elsewhere. Consequently, our findings may not be fully generalizable to all women with GDM in Nicaragua, particularly those in rural settings.

Fifth, the study's temporal coverage (September to December 2017) represents only one season, potentially missing seasonal variations in outcomes or care patterns. Finally, the retrospective nature of the study limited our ability to collect detailed information on important confounders such as socioeconomic status, diet quality, medication adherence, and glycemic control levels.

Despite these limitations, this study provides important insights into GDM outcomes in a previously understudied population and establishes groundwork for future prospective research in similar resource-constrained settings.

## Conclusions

This study highlights a high burden of maternal and fetal complications among women with GDM in Nicaragua, with obesity, advanced maternal age, and inadequate prenatal care emerging as key factors associated with outcomes such as preeclampsia, macrosomia, and cesarean delivery. Despite these individual complications, the overall clinical outcomes were largely favorable, suggesting resilience in maternal care systems even with resource constraints.

These findings underscore the need for locally adapted interventions, including enhanced screening strategies, improved prenatal care access, and standardized clinical guidelines tailored to low-resource settings. While not establishing causality, this study provides essential evidence to guide policy development, clinical practice, and future research aimed at reducing GDM-related morbidity in Nicaragua and similar settings.
